# Efficacy of language intervention in the early years

**DOI:** 10.1111/jcpp.12010

**Published:** 2012-11-23

**Authors:** Silke Fricke, Claudine Bowyer-Crane, Allyson J Haley, Charles Hulme, Margaret J Snowling

**Affiliations:** 1 Department of Human Communication Sciences, University of SheffieldSheffield; 2 Department of Psychology, Sociology and Politics, Sheffield Hallam UniversitySheffield; 3 Department of Psychology, University of YorkYork; 4 Division of Psychology and Language Sciences, University College LondonLondon, UK

**Keywords:** Intervention, language, mediation, reading, education

## Abstract

**Background:**

Oral language skills in the preschool and early school years are critical to educational success and provide the foundations for the later development of reading comprehension.

**Methods:**

In a randomized controlled trial, 180 children from 15 UK nursery schools (*n* = 12 from each setting; *M*_age_ = 4;0) were randomly allocated to receive a 30-week oral language intervention or to a waiting control group. Children in the intervention group received 30 weeks of oral language intervention, beginning in nursery (preschool), in three group sessions per week, continuing with daily sessions on transition to Reception class (pre-Year 1). The intervention was delivered by nursery staff and teaching assistants trained and supported by the research team. Following screening, children were assessed preintervention, following completion of the intervention and after a 6-month delay.

**Results:**

Children in the intervention group showed significantly better performance on measures of oral language and spoken narrative skills than children in the waiting control group immediately after the 30 week intervention and after a 6 month delay. Gains in word-level literacy skills were weaker, though clear improvements were observed on measures of phonological awareness. Importantly, improvements in oral language skills generalized to a standardized measure of reading comprehension at maintenance test.

**Conclusions:**

Early intervention for children with oral language difficulties is effective and can successfully support the skills, which underpin reading comprehension.

## Introduction

It is well established that learning to read builds on oral language skills. To become literate, children must develop the ability to decode print fluently and the skills to understand what they read; whereas decoding skills depend on phoneme awareness and letter knowledge, broader language skills are required for successful reading comprehension (e.g. Catts, Fey, Zhang, & Tomblin, 1999; Muter, Hulme, Snowling, & Stevenson, 2004). Moreover, oral language is a developmental precursor of phonological awareness (Burgess & Lonigan, 1998; Carroll, Snowling, Hulme, & Stevenson, 2003; Storch & Whitehurst, 2002). A corollary of this is that children with language difficulties typically have difficulties with literacy development (Pennington & Bishop, 2009, for review) and are at high risk of educational underachievement.

There is a growing body of evidence-based interventions for children with literacy difficulties (Fletcher, Reid Lyon, Fuchs, & Barnes, 2007; Snowling & Hulme, 2011; for reviews). These studies show that training in the alphabetic principle (letter-sound knowledge and phoneme awareness) combined with text reading are effective in improving word-level decoding difficulties (Hatcher, Hulme, & Snowling, 2004). However, there is variability in response to intervention with poor response being associated with severe phonological impairments (Nelson, Benner, & Gonzalez, 2003; Torgesen et al., 1999; Vellutino et al., 1996) or broader oral language difficulties, including poor vocabulary skills (Al Otaiba & Fuchs, 2006; Duff et al., 2008; Vadasy, Sanders, & Abbott, 2008; Whiteley, Smith, & Connors, 2007). Relatively few studies have targeted reading comprehension, but vocabulary instruction is known to benefit struggling readers (Elleman, Lindo, Morphy, & Compton, 2009) and broader oral language intervention has been shown to be effective for ‘poor comprehenders’ (Clarke, Snowling, Truelove, & Hulme, 2010).

Together the findings of these studies provide a rationale for interventions that promote oral language skills to build a secure foundation for literacy, but there is a dearth of evidence concerning their impact (cf. Bernhardt & Major, 2005; Hindson et al., 2005; Nancollis, Lawrie, & Dodd, 2005).

Munro, Lee, and Baker (2008) demonstrated improvements in phonological awareness, vocabulary and oral narrative following a hybrid language intervention targeting vocabulary knowledge and phonological awareness for 5-year-old children, but the lack of a control group limits the conclusions that can be drawn. Pollard-Durodola et al. (2011) randomly assigned 125 children to either receive shared book reading or practice as usual. Significant effects were found in favour of shared book reading, but these were restricted to experimental measures of vocabulary with no transfer to standardized measures, and there was no consideration of the impact of the intervention on literacy development.

Bowyer-Crane et al. (2008) identified children at school entry with poorly developed spoken language skills and provided these children with either an oral language intervention focusing on vocabulary and narrative skills, or a phonology with reading intervention focusing on letter-sound knowledge, phonological awareness and book reading. As expected, the phonology with reading intervention produced improvements in word-level reading skills. In contrast, the oral language intervention produced improvements in vocabulary knowledge and expressive grammar.

Similar findings were reported by Bianco et al. (2010) who compared three training programmes; one targeting components of language comprehension (e.g. monitoring, inference making), another targeting language comprehension skills implicitly via storybook reading and a phonological awareness programme. The programme training explicit comprehension skills produced significant improvements in spoken language comprehension, but not in phonological awareness skills, whereas phonological awareness training significantly improved children’s phonological skills, but not their comprehension. However, group allocation was not randomized, and no measure of transfer of gains to literacy development was included.

In summary, although it appears clear that language intervention can be implemented successfully with young children prior to, or at, school entry with positive results, the evidence-base for the effectiveness of oral language intervention starting in preschool is limited and the impact of such interventions on later literacy development remains unclear. The current study evaluated a 30-week language intervention programme delivered in the final term in Nursery school and the first two terms in Reception class. The intervention programme comprised activities targeting spoken language skills for the first 20 weeks, supplemented for the final 10 weeks with training in two critical components of the alphabetic principle, letter-sound knowledge and phoneme awareness. We predicted that children receiving the intervention programme would outperform an untreated control group on measures of language immediately after the intervention and there would be transfer to literacy skills, fostered by the work on phoneme awareness and letter-sound knowledge. We also followed the progress of a representative group of age-peers to compare progress of the intervention group against age norms.

## Methods

**Figure 1:**
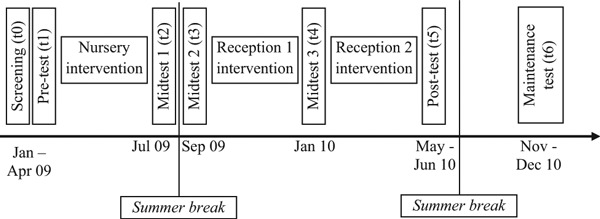
Time line of 30 weeks intervention and assessments

Following screening in 19 preschool settings to identify children with weak oral language skills, we conducted a randomized controlled trial in which children were allocated to either an oral language intervention or a waiting control group. Children allocated to the intervention group received support additional to ‘mainstream’ activities in a 30-week oral language programme beginning in Nursery in the term prior to formal school entry (10 weeks) and continuing in the Reception classroom for 20 weeks [The early education system in England is divided into Nursery (ages 3–4) and Primary school (ages 4–11; Reception and Years 1–6). The Early Years Foundation Stage (Nursery and Reception; with the Reception year being pre-Year 1) became part of the National Curriculum in 2002. By law, full-time education is compulsory for all children aged 5–16 and children begin full-time primary education in England by attending a Reception class in the school year they turn 5]. The ‘waiting’ group received no additional teaching during that time. Children’s performance was assessed at screening (t0) and pretest (t1) and progress was monitored throughout the intervention (t2–t5) and at a 6-month follow-up (t6). Herein, we report data from screening (t0), pretest (t1), immediate post-test (t5) and delayed follow-up (t6). The primary outcome measures were language and spoken narrative skills; the secondary outcomes were phoneme awareness and literacy skills. As a control measure, we also assessed gains in number skills, which were not targeted by the intervention. The timeline for assessments is presented in Figure 1.

Ethical permission for the study was granted by the Research Ethics Committee of the Department of Psychology, University of York. Head teacher consent was given for the intervention to be delivered in schools and informed parental consent was given for each child who participated in the study.

### Participants

**Figure 2:**
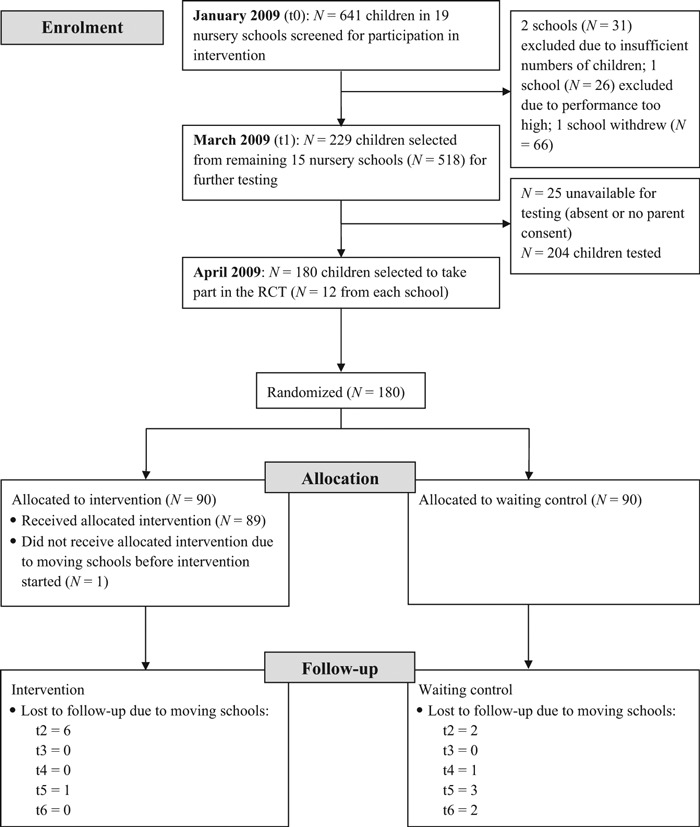
Flowchart showing details of selection and allocation of participants and flow through study

Details of recruitment, selection, allocation and the flow of participants through the study are summarized in Figure 2, in accordance with CONSORT guidelines (Schulz, Altman, & Moher, 2010). Nineteen Nursery schools in Yorkshire (England) were involved at the outset of the study. In these Nursery schools, all children who were due to enter school (Reception) in the following academic year were screened (t0). Following screening, one school withdrew from the study, two schools were deemed unsuitable for continued involvement due to insufficient numbers of children, and one school was excluded due to relatively high performance of their children on language measures.

In each of the remaining 15 nursery schools, approximately 15 children with the lowest mean verbal composite score were selected as possible participants in the trial. The verbal composite was based on *z*-scores on screening measures (*CELF* Preschool II^UK^*Recalling Sentences* and *Expressive Vocabulary* subtests (Semel, Wiig, & Secord, 2006) and the Early Repetition Battery *Word- and Nonword Repetition* subtests (Seeff-Gabriel, Chiat, & Roy, 2008). To validate this initial selection, individual language and literacy assessments were conducted with each of these children (*t1*; see below). The 12 children in each Nursery school (*N* = 180; *M*_age_ = 4;0) with the lowest scores on a composite measure derived from the following CELF Preschool II^UK^ subtests (*Recalling Sentences, Expressive Vocabulary, Sentence Structure, and Word Structure*) were selected to take part in the trial and randomly allocated to either the intervention (*N* = 90; six from each school) or waiting control groups (*N* = 90; six from each school). In addition, six children in each school matched on gender and date of birth to a random sample of three children from the intervention and the waiting control groups acted as a representative peer comparison group against which to benchmark the progress of children (*N* = 82).

### Assessment measures

The measures tapped skills directly targeted by the intervention and standardized measures of language and literacy. Due to time constraints, not all tests were administered at each testing point.

### Screening tests

The screening tests (*t0*) provided an assessment of children’s vocabulary and grammatical development using CELF Preschool II^UK^*Expressive Vocabulary* (requiring picture naming) and *Recalling Sentences* (requiring sentence repetition), *Early Repetition Battery* word and nonword repetition. Sentence repetition and nonword repetition are established markers of language impairment (Conti-Ramsden, Botting, & Faragher, 2001).

### Pre, mid, post and maintenance tests

#### Language skills

*Grammar:* Grammatical skills were assessed using several measures: CELF Preschool II^UK^*Sentence Structure* (*t1, t3, t5*) requires matching one of four pictures to a sentence. The Renfrew Action Picture Test (APT; Renfrew, 2003; *t1, t2, t4, t5, t6*) measures use of grammar (*Grammar Score*) and vocabulary (*Information Score*) for describing pictured actions. CELF Preschool II^UK^*Word Structure* (*t1, t5*) requires the production of inflected forms of verbs and nouns.

*Vocabulary:* Vocabulary knowledge was measured using the CELF Preschool II^UK^*Expressive Vocabulary* test (*t0, t4, t5, t6*).

*Listening comprehension:* Listening comprehension was assessed by children listening to two short stories and answering questions about them (*t1, t5, t6*).

*Narrative skills* were measured using a story retelling task (Squirrel Story; Carey, Leitao, & Allan, 2006; *t1, t2, t4, t5, t6*). Narratives were transcribed verbatim and analysed to derive three scores: mean length of utterance in words (MLUw), the number of words used (NW) and the number of different words used (NDW) retelling the story.

*Taught vocabulary:* The vocabulary taught in the Nursery intervention was assessed using *Expressive Picture Naming* and *Receptive Picture Selection* (*t1, t2, t4, t5*). The vocabulary taught in the Reception intervention was assessed using *Picture Naming* (*t5, t6*) and *Definitions* task (*t3, t4, t5, t6*).

#### Phonological awareness

*Onset awareness:* Measured using an *Alliteration Matching* task in which one of two pictures had to be matched to a target picture based on first sound (Carroll & Snowling, 2001). *Phoneme awareness:* Measured using YARC *Sound Isolation* in which the initial or final sound has to be identified from spoken non words (*t1, t3, t5*) and Sound Linkage *Segmentation, Blending and Deletion* tasks (*t6*; Hatcher, 2001).

#### Literacy skills

*Letter-sound knowledge:* Knowledge of letter-sounds was assessed at *t1, t2, t3, t4, t5, t6.*

*Reading*: The YARC *Early Word Reading* (Hulme et al., 2009) assessed single word reading (*t1, t3, t4, t5, t6*). Text reading accuracy and reading comprehension (*t6*) was assessed using the YARC beginner passage (Snowling et al., 2009).

*Spelling* was measured by giving children 5 (10) pictures to name and spell (*t3, t4, t5, t6*). Spelling responses were scored for number of consonants correct.

#### General cognitive ability

The *Block Design* subscale from the Wechsler Preschool and Primary Scale of Intelligence (WPPSI III UK, Wechsler, 2003) was used as a measure of nonverbal ability (*t2*).

### Intervention programme

Children allocated to the intervention group took part in a 30-week intervention programme delivered by teaching assistants selected by their nursery/school. The first 10 weeks involved three 15-min group sessions (2–4 children per group) per week delivered in preschool. Once the children entered school, this increased to three 30-min sessions plus two 15-min individual sessions. Children were taught using multisensory techniques within a standard framework; a detailed schedule for the group and individual sessions is presented in Table S1.

The oral language programme aimed to improve children’s vocabulary, develop narrative skills, encourage active listening and build confidence in independent speaking. The programme was a modified version of that shown to be effective by Bowyer-Crane et al. (2008), designed with reference to the UK’s Primary Framework for Literacy and Mathematics (DfES, 2006) and the Statutory Framework for the Early Years Foundation Stage (DCSF, 2008). New vocabulary was selected with reference to themes common in Early Years’ settings (e.g. Growing, Journeys, Ourselves and Time) and included nouns, verbs, adjectives, prepositions, pronouns and question words; taught using multisensory techniques (Beck, McKeown, & Kucan, 2002). Narrative work encouraged expressive language and grammatical competence. Activities revolved around creating and acting out stories, sequencing and story elements. Listening skills were specifically targeted in the first 20 weeks during the *Sound/Listening Game* incorporating ideas from *Letters and Sounds: Phase 1* (DfES, 2007). This section was extended in the last 10 weeks by activities to promote phoneme awareness (blending and segmenting) and letter-sound knowledge.

Teaching assistants received 2 days training prior to each 10-week block of intervention and attended fortnightly tutorials over the course of the programme. For each 10-week intervention block, a manual described activities and procedures, including materials and resources. To monitor treatment fidelity, teaching assistants attended regular tutorials and the research team observed each teaching assistant delivering intervention and provided feedback on five occasions. In addition, teaching assistants completed records of session plans, children’s progress and attendance for each group and individual session.

## Results

Descriptive statistics for all measures at screening (*t0*), pretest (*t1*), immediate post-intervention (after 30 weeks, *t5*) and maintenance test (6 months later, *t6*) are shown in Table 1 for the intervention and waiting control groups (data for the peer controls is given in Table S2). It can be seen that the intervention and control groups are approximately equated on all measures at t1, as expected given random assignment.

**Table 1:** Mean raw scores (*SD*) for intervention and waiting control groups for primary and secondary outcome measures at screening (t0), preintervention (t1), immediately postintervention (t5), and at delayed follow-up (t6; with effect sizes for intervention effects)

	Intervention			Waiting control		
			
	Reliability	*M*	*SD*	*M*	*SD*	Cohen’s *d*
Age (years;months)						
Screening t0	n/a	4;0	0;4	4;0	0;4	
Postintervention t5	n/a	5;3	0;4	5;3	0;3	
Delayed follow-up t6	n/a	5;8	0;4	5;9	0;3	
Screening/pretest only						
CELF-RS t0 (37)	.88^a^	7.26	5.58	7.56	5.99	
PSRep t0 (36)	.89^a^	26.02	6.41	26.94	5.33	
Primary outcomes						
CELF-EV						
t0 (40)	.82^a^	12.60	6.09	12.37	5.97	
t 5 (70)		32.16	10.02	27.84	9.60	.68^1^
t 6 (70)		36.27	8.54	32.17	9.14	.64^1^
CELF-SS						
t1 (22)	.78^a^	10.15	4.06	10.20	4.45	
t5 (34)		23.45	5.16	22.86	4.50	.15^1^
APT information						
t1 (40)	.98^b^	20.65	6.16	21.06	5.87	
t5		31.40	4.91	29.65	4.88	.36^1^
t6		31.37	4.73	28.90	5.08	.48^1^
APT grammar						
t1 (37)	.92^b^	12.09	5.41	14.44	5.26	
t5		24.60	5.43	22.05	5.71	.92^1^
t6		25.11	4.98	21.60	5.15	1.10^1^
Listening comprehension						
t1 (16)	.99^b^	3.05	2.43	3.14	2.99	
t5 (16)		6.41	3.34	5.59	3.33	.33^1^
t6 (14)		7.57	3.00	6.11	2.75	.57^1^
Narrative MLUw						
t1	.90^b^	4.28	1.96	4.74	1.66	
t5		6.81	2.16	6.79	1.78	.27^1^
t6		7.62	1.95	7.81	2.38	.15^1^
Narrative NW						
t1	.99^b^	50.50	32.77	55.25	34.80	
t5		102.81	47.97	86.58	38.57	.62^1^
t6		113.15	44.52	101.51	45.10	.48^1^
Narrative NDW						
t1	.99^b^	12.49	7.16	13.27	6.93	
t5		26.23	9.97	23.15	8.85	.55^1^
t6		27.36	8.86	24.42	9.68	.53^1^
Secondary outcomes						
Alliteration matching						
t1 (10)		3.72	2.31	4.31	2.18	
t5		7.17	2.28	6.59	2.28	.52^1^
Sound isolation						
t1 (12)	.88^a^	0.09	0.36	0.29	0.87	
t5		5.83	3.70	5.46	3.56	.13^2^
Segm/Blen/Del t6 (18)	.89^a^	8.42	4.11	7.55	4.32	.21^2^
Letter knowledge						
t1 (17)	.95^a^	1.36	1.70	1.35	2.35	
t5 (17)		13.62	3.68	12.50	3.53	.54^1^
t6 (32)		27.94	5.59	26.88	5.60	.51^1^
Early word reading						
t1 (30)	.98^a^	0.00	0.00	0.03	0.18	
t5		7.73	6.34	6.68	6.98	.16^2^
t6		11.94	7.03	11.57	8.73	.05^2^
Text reading accuracy (errors) t6	.75^c^	8.57	5.41	8.32	5.84	−.05^2^
Reading comprehension t6 (8)	.77^a^	4.80	1.58	3.91	1.83	.52^2^
Spelling						
t3 (68)	.95^a^	4.07	5.20	5.42	7.59	
t5 (68)		35.75	18.17	31.78	18.24	.82^1^
t6 (136)		70.86	30.21	69.94	32.44	.35^1^
General cognitive ability						
WPPSI block design	.84^d^	9.00	2.65	8.91	3.02	

(), maximum raw scores; RS, recalling sentences; EV, expressive vocabulary; SS, sentence structure; WS, word structure; APT, Action Picture Tests; MLUw, mean length of utterance words; NW, number of words; NDW, number of different words; WPPSI, Wechsler Preschool and Primary Scale of Intelligence.

Reliability: ^a^Cronbach’s alpha; ^b^Interrater reliability; ^c^Correlation between parallel test forms; ^d^Split-half reliability.

Cohen’s *d*: ^1^Difference in progress between groups divided by pooled initial *SD*; ^2^Difference in means divided by pooled *SD*; t3 Spelling scores given as baseline as not tested at t0 or t1.

All data analyses were conducted in Stata 12.0 (Stata Corp, College Station, Texas, USA) using either hierarchical linear models or structural equation models (SEM), using Maximum Likelihood Missing Value estimators to allow for missing data and robust (Huber–White) standard errors to allow for the clustering of children within schools. In the SEM models, effect sizes for the intervention effect were calculated from the *y*-standardized partial regression coefficients for the dummy-coded Group variable. These partial regression coefficients can be interpreted as equivalent to Cohen’s *d*; they express the difference in group means in *z*-score units after allowing for any group differences at baseline (Brown, 2006).

### Effects of intervention on directly taught skills

Table 2 shows the effects of the intervention on the vocabulary, which was taught in nursery (weeks 1–10) and reception (weeks 11–30) class and on the letter-sounds taught in weeks 21–30. It is clear that there were substantial effects of the intervention on vocabulary taught during weeks 11–30 (effect sizes 0.83–1.18), but much smaller effects on vocabulary taught during weeks 1–10 (effect sizes 0.25–0.27). The intervention also appeared successful in teaching children letter-sounds during weeks 21–30 (effect size 0.41). The *z* values and significance levels for the hierarchical linear models (Table 2) confirmed that there were no significant effects of the intervention on vocabulary taught during weeks 1–10 (*Nursery: Expressive naming* and *Nursery: Receptive*). However, there were highly significant effects of the intervention on t5 and t6 *Reception: Expressive Naming*, t5 *Letter knowledge,* and t6 *Reception: Definitions.*

**Table 2:** Mean raw scores (*SD*) for intervention and waiting control groups preintervention, immediately postintervention (t5) and at delayed follow-up (t6) for measures, which were directly taught (with effect sizes)

		Intervention		Waiting Control			HLM	
					
	Reliability	*M*	*SD*	*M*	*SD*	Cohen’s *d*	*z*	*p*
Intervention vocabulary								
Nursery: Expressive naming								
t1 (12)	.67^a^	1.43	1.36	1.43	1.23			
t5		4.83	2.21	4.48	2.07	.27^1^	1.41	.158
Nursery: Receptive								
t1 (12)	.66^a^	5.64	1.82	5.64	2.16			
t5		9.04	1.75	8.54	2.08	.25^1^	1.58	.114
Reception: Definitions								
t3 (84)	.99^b^	12.90	6.26	13.47	6.98			
t5		29.13	9.13	21.87	7.46	1.18^1^	7.17	<.001
t6		29.68	8.15	23.12	6.33	1.08^1^	6.87	<.001
Reception: Expressive naming								
t5 (24)	.67^a^	16.94	3.97	13.72	3.79	.83^2^	6.80^3^	<.001
t6		17.87	2.76	14.23	3.71	1.11^2^	9.24^3^	<.001
Intervention letter knowledge								
t4 (32)	.91^a^	18.22	10.30	18.58	8.88			
t5		27.80	5.71	24.18	7.35	.41^1^	4.68	<.001

Reliability: ^a^Cronbach’s alpha; ^b^Interrater reliability.

Cohen’s *d*: ^1^Difference in progress between groups divided by pooled initial *SD*; ^2^Difference in means divided by pooled *SD*; HLM: autoregressor entered as covariate, ^3^t0 CELF EV entered as autoregressor; t3 Reception: Definitions scores and t4 Intervention letter knowledge scores given as baseline as not tested at t0 or t1.

### Generalization to standardized tests

**Figure 3:**
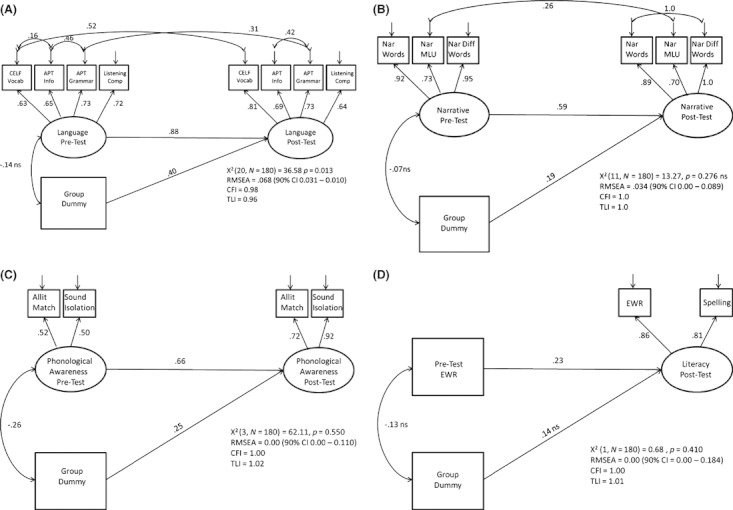
Models showing the effects of the intervention on Language (A), Narrative (B), Phonological Awareness (C) and Literacy (D) skills at immediate post-test (t5)

**Figure 4:**
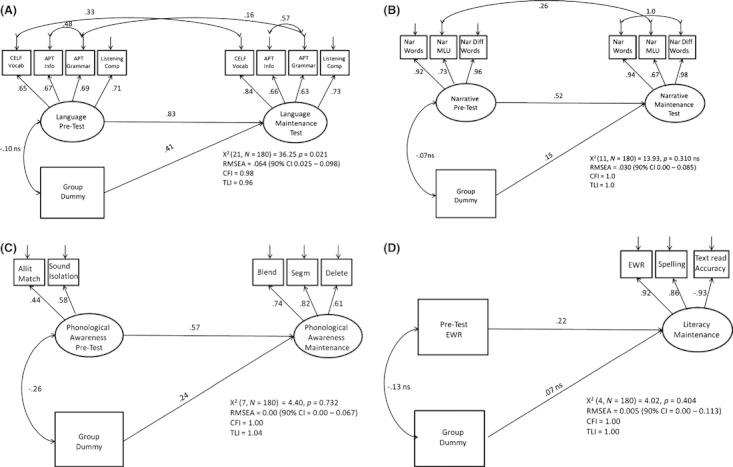
Models showing the effects of the intervention on Language (A), Narrative (B), Phonological Awareness (C) and Literacy (D) skills at delayed maintenance test (t6)

Our principal interest was to examine the extent to which the intervention produced generalized improvements on tests assessing primary outcomes in the domains of language, and narrative skills and on secondary outcomes, phoneme awareness and literacy skills (see Table 1). To assess these effects we constructed latent variable models in which the four constructs (Language, Narrative, Phoneme Awareness, Literacy) were defined at pretest (*t1*) immediate post-test (*t5*) and maintenance follow-up (*t6*) by multiple measures. The measures used to define each construct at each point in time are shown in the Path diagrams representing these models (Figures 3 and 4). The effects of these analyses were remarkably clear. At immediate and delayed post-test, there were significant effects of the intervention on Language (immediate post-test *d* = .80, *z* = 6.57, *p* < .001; maintenance test *d* = .83, *z* = 2.41, *p* < .001), Narrative (immediate post-test *d* = .39, *z* = 2.97; *p* = .003 maintenance test *d* = .30, *z* = 2.04, *p* = .041) and Phoneme Awareness (immediate post-test *d* = .49, *z* = 2.16, *p* = .031; maintenance test *d* = .49, *z* = 2.58; *p* = .01). The effects on Literacy were not significant (immediate post-test *d* = .31; *z* = 1.81; *p* = .07; maintenance test *d* = .14, *z* = .93, *p* = .354).

Finally, it is important to note that the intervention group showed higher scores on a reading comprehension test that was first administered at maintenance follow-up. With baseline word-reading skill as the covariate, in a hierarchical linear model with school as a fixed effect, there was a highly reliable effect of the intervention (marginal mean group difference = 0.97, 95% CI 0.40–1.54, *z* = 3.32, *p* = .001). It should also be noted that this effect remained essentially unchanged when reading accuracy on the reading comprehension test at maintenance test was an additional covariate (marginal mean group difference = 0.91, 95% CI 0.42–1.41, *z* = 3.63, *p* < .001). Thus, the effect of the intervention on reading comprehension is not accounted for by differences in reading accuracy, but appears to be a generalized effect from the improvements in language comprehension skill brought about by the intervention.

**Figure 5:**
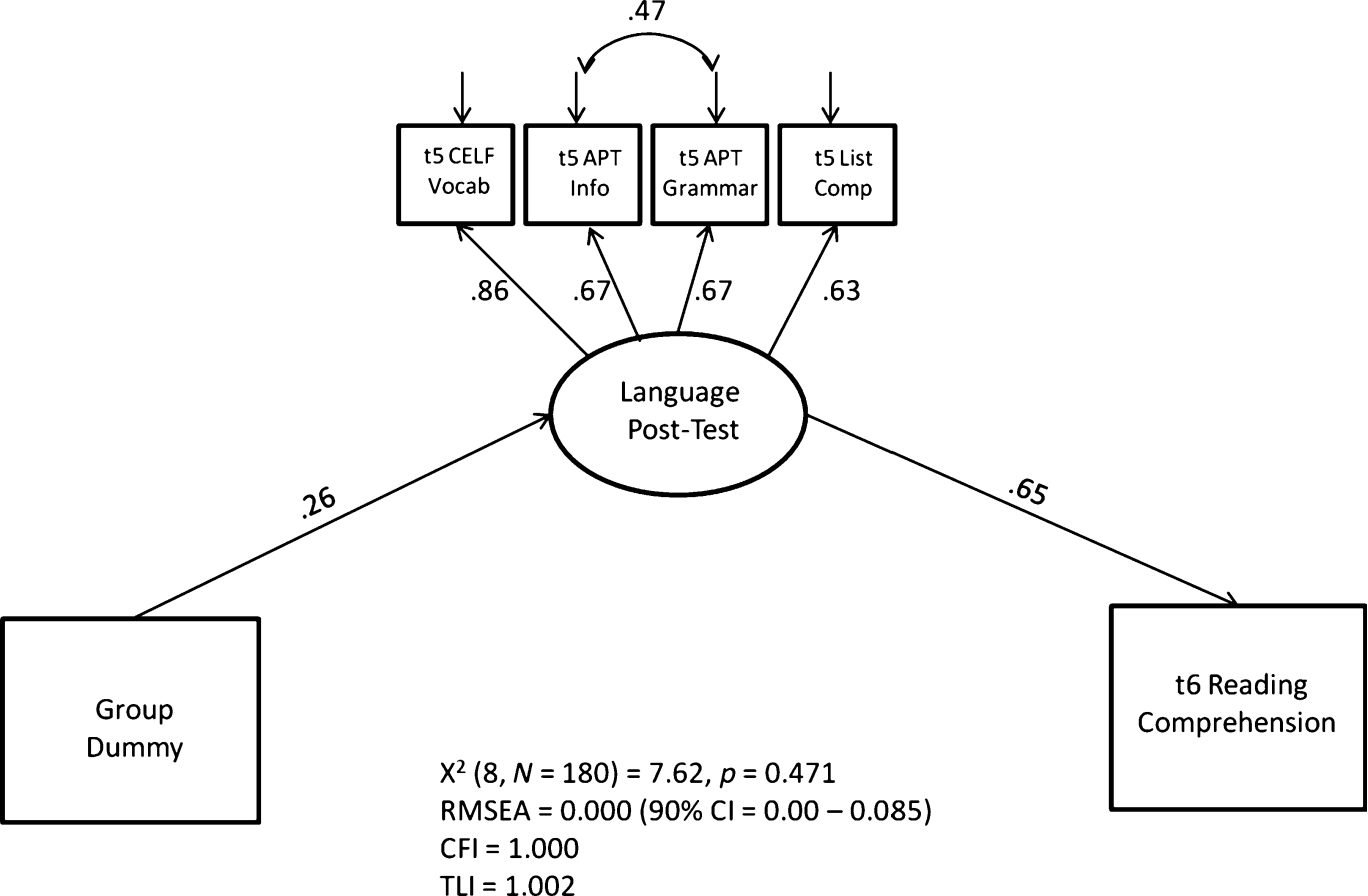
Mediation model showing the effect of intervention on reading comprehension outcome at t6 as product of improvements in oral language abilities at t5

To test this hypothesis, we ran a mediation model in which the effect of intervention group on reading comprehension outcome at t6 was the product of improvements in language comprehension abilities at t5 (see Figure 5). In this model, there is complete mediation, with the effect of intervention group on reading comprehension scores being accounted for entirely by the indirect pathway (intervention group → language comprehension t5 → reading comprehension t6). Dropping the direct pathway (intervention group → reading comprehension t6) had no significant effect on the fit of the model (χ^2^ difference = 0.37, df = 1, NS). The compound path for the indirect effect (intervention group → language comprehension t5 → reading comprehension t6) is highly significant (*z* = 23.68, *p* < .001).

To check for possible ‘Hawthorne’ or expectancy effects, arithmetic performance using the BAS Number Concept subtest before and after the intervention was assessed. In a hierarchical linear model with school as a fixed effect, there was no trace of any effect of the intervention on this measure (marginal mean group difference = 0.23, 95% CI −0.45 to 0.90, *z* = 0.66, *p* = .511).

Table S1 reports data comparing the children in the intervention and waiting control group to the peer comparison group at immediate post-test and maintenance test. These figures allow assessment of the extent to which the intervention had improved language skills to levels comparable to a representative sample of peer controls from the same classrooms. For the measures of language taken at screening and pretest (*CELF Recalling Sentences*, *Word- and Nonword Repetition*, *CELF Expressive Vocabulary*, *Renfrew Action Picture Test grammar* and *information*), the differences between the intervention group and the representative peer comparison group are extremely large (*d*s range from 1.10 to 1.65). It is clear that where the same measures were reassessed following the intervention, the corresponding group differences have reduced in size substantially (*d*s range from .23 to .53) For the remaining tests, for which baseline data are not available, the peer comparison group always show higher scores, but with small-to-medium effect sizes (*d*s range from .72 to .01). Overall looking at the standard scores and scaled scores shown in Table S1 it is clear that the intervention group are showing essentially normal scores (similar to those of the peer comparison group) on some measures (*CELF Expressive Vocabulary*, *Letter knowledge*), whereas on some other measures (*CELF Sentence Structure* and *Early Word Reading*) both the intervention and waiting list control group appear to continue to lag behind the peer comparison group.

Hierarchical linear models showed that on a number of tests the intervention group did not differ significantly from peer controls following intervention (*t6 APT information*: marginal mean group difference = −1.28, 95% CI −2.58 to 0.02, *z* = −1.93, *p* = .053; *t6 APT grammar*: marginal mean group difference = −1.06, 95% CI −2.45 to 0.33, *z* = −1.50, *p* = .134; *t5 letter knowledge*: marginal mean group difference = −.48, 95% CI −1.47 to 0.51, *z* = −0.95, *p* = .342; *t6 letter knowledge*: marginal mean group difference = −0.89, 95% CI −2.39 to 0.61, *z* = −1.17, *p* = .243; *t6 listening comprehension*: marginal mean group difference = −0.79, 95% CI −1.60 to 0.01, *z* = −1.94, *p* = .052; *t5 narrative NW*: marginal mean group difference = −0.46, 95% CI −13.45 to 12.52, *z* = −0.07, *p* = .944; *t5 narrative NDW*: marginal mean group difference = −1.49, 95% CI −4.30 to 1.32, *z* = −1.04, *p* = .299; *t6 reading comprehension*: marginal mean group difference = −0.28, 95% CI −0.88 to 0.32, *z* = −0.91, *p* = .364).

## Discussion

This study evaluated the effectiveness of an intervention designed to improve the oral language and early literacy skills of children identified in preschool as having poorly developed language. Participating children were randomly assigned to an oral language intervention or a waiting control group. The intervention programme focused primarily on vocabulary, narrative and listening skills, with additional work on letter-sound knowledge and phoneme awareness introduced in the final 10 weeks. The intervention programme was effective in improving oral language and spoken narrative skills immediately following the intervention, and these effects were each maintained 6 months later.

There was evidence that the two preliteracy skills directly taught (letter-sound knowledge and phoneme awareness) benefitted from the intervention. However, these effects did not generalize to measures of word-level literacy skills. This may not be surprising given that these preliteracy skills were only introduced in the last 10 weeks of the programme, and such forms of training are known to be more effective when combined with reading instruction (Hatcher, Hulme, & Ellis, 1994; NRP, 2000).

Notwithstanding the absence of statistically reliable effects of the intervention on word-level literacy skills, a very encouraging outcome was the significant impact of the intervention on reading comprehension measured some 6 months after the intervention had ceased. It is also notable that this effect was statistically independent of any concurrent effects on prose reading accuracy, and the results of a mediation model support the conclusion that this effect is a product of the improved language comprehension skills that were fostered by the intervention (see also Clarke et al., 2010). Furthermore, it is noteworthy that the intervention group was performing at a comparable level to their peers in reading comprehension after receiving the intervention. Arguably, therefore the programme had secured a foundation for reading comprehension in its recipients. More generally, the findings suggest that oral language intervention can make educationally significant improvements to the language skills of children who are ‘at risk’ of failure and it is encouraging that, on the majority of measures, the intervention group had ‘closed the gap’ between themselves and the peer control group after the intervention was completed.

Together the findings of the current trial are very encouraging because the direct effects of the training the children received transferred to standardized measures (cf. Pollard-Durodola et al., 2011). Whereas Elleman et al. (2009) suggested in their meta-analysis of the impact of vocabulary instruction on reading comprehension that standardized measures are often insensitive to vocabulary growth, in this study, its effects were observed in spoken narrative as well as on formal vocabulary measures. However, our data also suggest that the success of vocabulary instruction might depend upon the age at which it is given or the amount of training received: there was no evidence that vocabulary instruction in preschool was effective, although the small effect sizes need to be interpreted cautiously given that exposure to new vocabulary was limited to three sessions per week in the first 10 weeks, compared with daily sessions in the second 20 weeks of the programme when the children were in school. In addition, all teaching in the Nursery part of the programme was in group sessions. It is plausible that the combination of group and individual sessions received during Reception may have been more effective, giving teaching assistants the opportunity to tailor their vocabulary instruction to meet the needs of individual children.

## Conclusion

The study has shown that oral language skills can be promoted during the early school years and that this produces effects that generalize to standardized tests of oral language skills and reading comprehension. The study is one of the first to deliver language intervention during the transition from preschool to primary school; a limitation of the design is that it is not possible to evaluate the advantage of starting early (as compared with at school entry) and the optimal timing for and duration of such interventions remain unknown. Nonetheless, the study provides further evidence that school-based oral language interventions can be successfully delivered by trained and supported school staff, a vital step towards developing evidence-based, cost-effective interventions that can be applied effectively in everyday school settings (Savage, Carless, & Erten, 2009). Research is badly needed to extend the current study to evaluate the efficacy of longer term intervention programmes for children with persisting language learning impairments.

**Key points**

- Children who enter school with poorly developed language skills are at high risk of educational failure.
- Data from an RCT show that a 30-week intervention delivered in the last 10 weeks of preschool and continuing for 20 weeks through the transition into primary education can improve oral language, vocabulary and narrative skills.
- Supplementing the language work with training in letter-sound knowledge and phoneme awareness for the final 10 weeks brings about gains in these skills.
- Children who received the intervention showed gains in reading comprehension mediated by gains in oral language (and not by gains in word-level reading skills).

## Correspondence

Silke Fricke, Department of Human Communication Sciences, University of Sheffield, 31 Claremont Crescent, Sheffield, S10 2TA, UK; Email: s.fricke@sheffield.ac.uk

## Supporting information

**Table S1.** Group and individual session schedule for oral language intervention programme in Nursery and Reception class.

**Table S2.** Mean raw scores, standard scores and scaled scores (*SD*s) for peer control, intervention and waiting control groups at screening, pre-intervention, immediately post-intervention (t5), and at delayed follow-up (t6) for keymeasures (with effect sizes, *z* values and significance levels from an HLM analysis).
