# Supplementary material for: Efficacy of language intervention in the early years
Source: J Child Psychol Psychiatry. 2012 Nov 23;54(3):280–90. doi: 10.1111/jcpp.12010 (PMC3593174; doi:10.1111/jcpp.12010)
Supplement: Supplementary file 1 [file jcpp0054-0280-SD1.docx]

Online supporting information for Fricke et al. Efficacy of language intervention in the early years. doi:10.1111/jcpp.12010

Table S1

*Group and individual session schedule for oral language intervention programme in Nursery and Reception class*

| Nursery (10 weeks) |  | Reception Part 1 (10 weeks) | |  | Reception Part 2 (10 weeks) | |
| --- | --- | --- | --- | --- | --- | --- |
| Group session (15 mins) |  | Group session (30 mins) | Individual session (15 mins) |  | Group session (30 mins) | Individual session (15 mins) |
| Introduction (3 mins) |  | Introduction (4 mins) | Introduction (2 mins) |  | Introduction (2 mins) | Introduction (1 min) |
|  |  |  |  |  | Letter sounds and phonological awareness (5 mins) | Letter sounds and phonological awareness (4mins) |
| Vocabulary Teaching (4 mins) |  | Vocabulary Revision (6 mins) | Vocabulary Revision (5 mins) |  | Vocabulary Revision (5 mins) | Vocabulary Revision (5 mins) |
|  |  | Vocabulary Teaching (6 mins) |  |  | Vocabulary Teaching (5 mins) |  |
| Narrative (5 mins) |  | Narrative (11 mins) | Narrative (5 mins) |  | Narrative (10 mins) | Narrative (4 mins) |
| Plenary (3 mins) |  | Plenary (3 mins) | Plenary (3 mins) |  | Plenary (3 mins) | Plenary (1 min) |

Table S2

*Mean raw scores, standard scores and scaled scores (SDs) for Peer Control, Intervention and Waiting Control Groups at screening, pre-intervention, immediately post-intervention (t5), and at delayed follow-up (t6) for key measures (with effect sizes, z values and significance levels from an HLM analysis).*

|  |  | Peer Control (PC) | |  | Intervention (IV) | |  | Waiting Controls (WC) | |  | PC vs. IV |  | PC vs. WC |  | HLM  PC vs. IV | |  | HLM  PC vs. WC | |
| --- | --- | --- | --- | --- | --- | --- | --- | --- | --- | --- | --- | --- | --- | --- | --- | --- | --- | --- | --- |
|  |  | *M* | SD |  | *M* | SD |  | *M* | SD |  | *d* |  | *d* |  | z | p |  | z | p |
| **Language measures** |  |  |  |  |  |  |  |  |  |  |  |  |  |  |  |  |  |  |  |
| CELF-RS t0 (37) |  | 15.79  ^1^(10.58) | 7.31  (2.22) |  | 7.26  (7.19) | 5.58  (2.76) |  | 7.56  (7.41) | 5.99  (2.59) |  | -1.31 |  | -1.23 |  |  |  |  |  |  |
| PSRep t0 (36) |  | 32.19  ^2^(103.26) | 2.56  (11.21) |  | 26.02  (82.28) | 6.41  (18.93) |  | 26.94  (84.99) | 5.33  (16.92) |  | -1.26 |  | -1.25 |  |  |  |  |  |  |
| CELF-EV   - t0 (40) - t 5 (70) - t 6 (70) |  | 19.53  ^1^(10.68)  37.81  ^1^(11.41)  40.72  ^1^(11.00) | 6.55  (2.60)  8.50  (2.31)  8.18  (2.51) |  | 12.60  (7.84)  32.16  (10.10)  36.27  (9.98) | 6.09  (2.64)  10.02  (2.79)  8.54  (2.54) |  | 12.37  (7.71)  27.84  (9.06)  32.17  (8.49) | 5.97  (2.93)  9.60  (2.61)  9.14  (2.39) |  | -1.10  -.61  -.53 |  | -1.14  -1.10  -.99 |  | 2.07  1.36 | .039  .175 |  | -2.32  -1.71 | .020  .088 |

*Continued over …/*

*Table S2 Continued /…*

| CELF-SS t5 (34) |  | 26.60  ^1^(10.68) | 3.36  (2.19) |  | 23.45  (8.94) | 5.16  (2.84) |  | 22.86  (8.50) | 4.50  (2.17) |  | -.72 |  | -.94 |  |  |  |  |  |  |
| --- | --- | --- | --- | --- | --- | --- | --- | --- | --- | --- | --- | --- | --- | --- | --- | --- | --- | --- | --- |
| APT information   - t1 (40) - t5 - t6 |  | 29.44  33.33  32.60 | 4.33  2.99  3.79 |  | 20.65  31.40  31.37 | 6.16  4.91  4.73 |  | 21.06  29.65  28.90 | 5.87  4.88  5.08 |  | -1.65  -.47  -.29 |  | -1.62  -.91  -.83 |  | 1.10  1.21 | .273  .225 |  | -2.54  -2.01 | .011  .045 |
| APT grammar   - t1 (37) - t5 - t6 |  | 20.05  26.21  26.17 | 4.56  4.26  4.03 |  | 12.09  24.60  25.11 | 5.41  5.43  4.98 |  | 14.44  22.05  21.60 | 5.26  5.71  5.15 |  | -1.59  -.33  -.23 |  | -1.14  -.83  -.99 |  | 3.20  2.01 | .003  .045 |  | -1.95  -3.63 | .051  <.001 |
| Listening comprehension   - t5 (16) - t6 (14) |  | 8.44  8.39 | 3.55  2.33 |  | 6.41  7.57 | 3.34  3.00 |  | 5.59  6.11 | 3.33  2.75 |  | -.59  -.31 |  | -.83  -.89 |  |  |  |  |  |  |
| Narrative MLUw   - t5 - t6 |  | 7.45  8.58 | 1.67  2.09 |  | 6.81  7.62 | 2.16  1.95 |  | 6.79  7.81 | 1.78  2.38 |  | -.33  -.47 |  | -.39  -.34 |  |  |  |  |  |  |

*Continued over …/*

*Table S2 Continued /…*

|  |  | Peer Control (PC) | |  | Intervention (IV) | |  | Waiting Controls (WC) | |  | PC vs. IV |  | PC vs. WC |  | HLM  PC vs. IV | |  | HLM  PC vs. WC | |
| --- | --- | --- | --- | --- | --- | --- | --- | --- | --- | --- | --- | --- | --- | --- | --- | --- | --- | --- | --- |
| Narrative NW   - t5 - t6 |  | 103.10  129.90 | 46.02  48.87 |  | 102.81  113.15 | 47.97  44.52 |  | 86.58  101.51 | 38.57  45.10 |  | -.01  -.36 |  | -.39  -.60 |  |  |  |  |  |  |
| Narrative NDW   - t5 - t6 |  | 27.86  31.03 | 9.60  10.22 |  | 26.23  27.36 | 9.97  8.86 |  | 23.15  24.42 | 8.85  9.68 |  | -.17  -.38 |  | -.68  -.66 |  |  |  |  |  |  |
| **Literacy-related measures** |  |  |  |  |  |  |  |  |  |  |  |  |  |  |  |  |  |  |  |
| Alliteration Matching t5 (10) |  | 7.97 | 2.02 |  | 7.17 | 2.28 |  | 6.59 | 2.28 |  | -.37 |  | -.64 |  |  |  |  |  |  |
| Sound Isolation t5 (12) |  | 7.17 | 2.94 |  | 5.83 | 3.70 |  | 5.46 | 3.56 |  | -.38 |  | -.53 |  |  |  |  |  |  |
| Segm/Blen/Del t6 (18) |  | 10.71 | 3.84 |  | 8.42 | 4.11 |  | 7.55 | 4.32 |  | -.58 |  | -.77 |  |  |  |  |  |  |
| Letter Knowledge   - t1 (17) - t5 (17) - t6 (32) |  | 2.46  ^2^(101.04)  14.11  ^2^(110.88)  28.89  ^2^(106.21) | 2.89  (15.71)  3.39  (14.42)  4.40  (18.49) |  | 1.36  (96.19)  13.62  (109.68)  27.94  (106.29) | 1.70  (11.59)  3.68  (13.22)  5.59  (14.23) |  | 1.35  (94.20)  12.50  (104.57)  26.88  (102.56) | 2.35  (13.27)  3.53  (14.39)  5.60  (14.15) |  | -.46  -.14  -.19 |  | -.42  -.46  -.40 |  | 0.24  0.01 | .814  .995 |  | -1.98  -1.56 | .047  .118 |

*Continued over …/*

*Table S2 Continued /…*

| Early Word Reading   - t5 (30) - t6 |  | 10.36  ^2^(106.01)  15.79  ^2^(100.88) | 7.56  (15.85)  8.60  (17.84) |  | 7.73  (102.37)  11.94  (97.67) | 6.34  (15.38)  7.03  (14.53) |  | 6.68  (99.07)  11.57  (96.95) | 6.98  (15.80)  8.73  (15.48) |  | -.38  -.49 |  | -.51  -.49 |  |  |  |  |  |  |
| --- | --- | --- | --- | --- | --- | --- | --- | --- | --- | --- | --- | --- | --- | --- | --- | --- | --- | --- | --- |
| Text Reading Accuracy (errors) t6 |  | 6.53 | 5.43 |  | 8.57 | 5.41 |  | 8.32 | 5.84 |  | .38 |  | .32 |  |  |  |  |  |  |
| Reading Comprehension t6 (8) |  | 5.08 | 1.86 |  | 4.80 | 1.58 |  | 3.91 | 1.83 |  | -.16 |  | -.63 |  |  |  |  |  |  |
| Spelling   - t3 (68) - t5 (68) - t6 (136) |  | 9.07  43.57  90.64 | 9.72  13.98  24.83 |  | 4.07  35.75  70.86 | 5.20  18.17  30.21 |  | 5.42  31.78  69.94 | 7.59  18.24  32.44 |  | -.64  -.48  -.71 |  | -.42  -.73  -.72 |  | -1.61  -3.33 | .107  .001 |  | -3.96  -3.80 | <.001  <.001 |

*Note.* ( ) = Maximum raw scores; RS = Recalling Sentences; EV = Expressive Vocabulary; SS = Sentence Structure; WS = Word Structure; APT = Action Picture Tests; MLUw = Mean Length of Utterance Words; NDW = Number of Different Words; Cohen’s d: difference in means divided by pooled SD; HLM: conducted with random intercept and fixed slopes and the autoregressor entered as covariate; 1 = Scaled Scores for CELF Preschool II^UK^ subtests; 2 = Standard Scores for Early Repetition Battery, YARC Letter Sound Knowledge and YARC Early Word Reading
